# Preparation and Antimicrobial Action of Three Tryptic Digested Functional Molecules of Bovine Lactoferrin

**DOI:** 10.1371/journal.pone.0090011

**Published:** 2014-03-03

**Authors:** Nilisha Rastogi, Nitish Nagpal, Hammad Alam, Sadanand Pandey, Lovely Gautam, Mau Sinha, Kouichirou Shin, Nikhat Manzoor, Jugsharan S. Virdi, Punit Kaur, Sujata Sharma, Tej P. Singh

**Affiliations:** 1 Department of Biophysics, All India Institute of Medical Sciences, New Delhi, India; 2 Department of Microbiology, University of Delhi, South Campus, New Delhi, India; 3 Department of Biosciences, Jamia Milia Islamia, New Delhi, India; 4 Food Science & Technology Institute, Morinaga Milk Industry Co. Ltd., Zama, Kanagawa, Japan; University Hospital Schleswig-Holstein, Campus Kiel, Germany

## Abstract

Lactoferrin is an 80 kDa bilobal, iron binding glycoprotein which is primarily antimicrobial in nature. The hydrolysis of lactoferrin by various proteases in the gut produces several functional fragments of lactoferrin which have varying molecular sizes and properties. Here, bovine lactoferrin has been hydrolyzed by trypsin, the major enzyme present in the gut, to produce three functional molecules of sizes approximately 21 kDa, 38 kDa and 45 kDa. The molecules have been purified using ion exchange and gel filtration chromatography and identified using N-terminal sequencing, which reveals that while the 21 kDa molecule corresponds to the N2 domain (21LF), the 38 kDa represents the whole C-lobe (38LF) and the 45 kDa is a portion of N1 domain of N-lobe attached to the C-lobe (45LF). The iron binding and release properties of 21LF, 38LF and 45LF have been studied and compared. The sequence and structure analysis of the portions of the excision sites of LF from various species have been done. The antibacterial properties of these three molecules against bacterial strains, *Streptococcus pyogenes*, *Escherichia coli*, *Yersinia enterocolitica* and *Listeria monocytogenes* were investigated. The antifungal action of the molecules was also evaluated against *Candida albicans*. This is the first report on the antimicrobial actions of the trypsin cleaved functional molecules of lactoferrin from any species.

## Introduction

Lactoferrin (LF) is a potent antimicrobial iron-binding glycoprotein found in colostrum [1]–[3] and other exocrine mammalian secretions like milk, tears, nasal secretions, saliva, amniotic fluid, urine and uterine secretions [4]–[9]. It is responsible for the defense and the development of the neonate [10], [11]. Structurally, LF is an 80 kDa monomer which contains two equal monoferric lobes, the N-lobe and the C-lobe [12]–[17]. Both the lobes are connected to each other by a short 3_10_-helix. Each lobe contains two equal domains, named as N1 and N2 in N-lobe and C1 and C2 in the C-lobe [18], [19].

LF has been known to show a broad spectrum antimicrobial activity against variety of bacteria, viruses and fungi in vitro [20]–[22]. It has been observed that LF exerts its antimicrobial action through iron sequestration as a native molecule. However, since LF is exposed to various proteases in the gut and subsequently, gets cleaved into various functional fragments, it would be relevant to study the antimicrobial effect as well as the iron release properties of these hydrolyzed molecules.

The first report of hydrolysis of LF by trypsin appeared in 1976 where iron-saturated and iron-free forms of LF and transferrin were subjected to limited proteolysis by trypsin which yielded five different LF fragments with molecular weights ranging from 25 to 52 kDa. It was also found that the iron-free forms of both proteins were more susceptible to hydrolysis proteins. In another study, a comparison of antimicrobial activity and iron-binding capacity of bovine lactoferrin before and after proteolysis with trypsin and chymotrypsin was done [23].It was observed that the enzymes did not have any significant effect on the antimicrobial activity or iron binding capacity of lactoferrin. In yet another related study, N- and C-terminal half molecules were produced using trypsin and chymotrypsin in the presence of urea [24]. Legrande et al demonstrated the generation of two iron-binding LF fragments of 30 kDa and 50 kDa using a milder treatment with trypsin [25]. Subsequently, the C-lobe of LF was also produced using trypsin [26]. All the above studies focused only on the preparation of LF fragments.

Though a number of reports on the antimicrobial effect of pepsin hydrolyzates of LF are present [27]–[30], there are no studies on the antimicrobial action of LF hydrolyzates produced by trypsin. Also, it has been established that peptides from the N1 domain of LF which are generated upon hydrolysis by proteases are very potent antimicrobial agents [31]–[34].

Here, we have isolated and purified three high molecular weight protein fragments produced by proteolysis of LF by trypsin. We have further compared the iron release properties of these three molecules with native LF. The sequence and the structural properties of the proteolysis sites of LF from various species have been characterized. The antimicrobial properties of these molecules against four different bacterial strains, *Streptococcus pyogenes* (*S. pyogenes*), *Listeria monocytogenes* (*L. monocytogenes*), *Escherichia coli* (*E. coli*), and *Yersinia enterocolitica* (*Y. enterocolitica*) and fungal strain of *Candida albicans (C. albicans)* have been characterized.

## Materials and Methods

### Preparation of trypsin digested fragments of Bovine LF

Bovine LF was obtained from Morinaga (Tokyo, Japan). The lyophilized samples of LF were dissolved in 50 mM Tris-HCl with 0.02 M CaCl_2_ at pH 7.8 and incubated with trypsin at a protein: enzyme molar ratio of 30∶1 for 1.5 h at 37°C as described by Brock et al. [35]. After 1.5 h of incubation, trypsin was neutralized by the addition of 0.035 M benzamidine. The molecular masses of the major fragments in the hydrolyzate were estimated using sodium dodecyl sulphate-polyacrylamide gel electrophoresis (SDS-PAGE).

### Purification of tryptic digested fragments of Bovine LF

The hydrolyzed product of bovine LF was passed through a cation exchanger CM Sephadex C-50 column (10 cm ×2.5 cm) with a salt gradient of (0.0–0.5) M NaCl in 50 mM Tris-HCl at pH 8.0. The unbound and the bound fractions were eluted, dialyzed and passed separately through a gel filtration column (100 cm ×2 cm) of Sephadex G-75 in 50 mM Tris-HCl, pH 8.0. All the peaks obtained after gel filtration were pooled, dialyzed against deionized water, lyophilized and stored at 216K. The molecular masses of the purified peaks were determined using SDS-PAGE.

### N-terminal sequencing

To identify the fragments obtained from hydrolysis of LF, N-terminal sequencing of all the three purified fragments was done by Edman degradation using PPSQ 21-A protein sequencer (Shimadzu, Japan).

### Sequence and structure analysis of proteolysis sites

In order to analyze and compare the sites in LF from various species which are cleaved by trypsin, the sequence alignment of the LFs from different species was done using ClustalW [36]. Similarly, the structures of these sites were compared with the program Pymol [37] using the relevant PDBs, 1BLF (bovine LF or CLF), 1B0L (human LF or HLF), 1BIY (buffalo LF or BLF), 1B7U (equine LF or ELF) and 1 JW1 (caprine LF or GLF).

### Iron saturation of native LF and the tryptic fragments

Iron saturation of the molecules was performed by the procedure as described by Mazurier and Spik [38]. 2 mM ferric chloride hexahydrate (FeCl_3_.6H_2_O) reagent was prepared in 0.1 M sodium bicarbonate/0.1 M sodium citrate buffer (pH 8.6). 1 ml of 1 mM protein solution was prepared in 0.1 M sodium bicarbonate/0.1 M sodium citrate buffer (pH 8.6) and further equilibrated with 1.2 ml of ferric chloride hexahydrate reagent for 16 h at 298 K. Excess of the ferric chloride reagent was removed from the preparation by dialyzing it against distilled water.

### Preparation of apo-forms of LF, 21LF, 38LF and 45LF

The apo-forms of LF and its three fragments were obtained by removing iron from the saturated purified molecules using the method as described by Masson et al. [39]. Each of the protein solutions (1%) dissolved in 50 mM Tris-HCl, pH 8.0 was dialyzed against an excess of 0.1 M citric acid for 24 h with regular changes after every 6 h and at different values of pH with intervals of 1 unit, starting from pH 8.5 to pH 2.0. The extent of iron saturation in the molecules was evaluated by estimating the iron saturation at every pH obtained from the ratio of absorbance A_465_/A_280_. At the end, the citric acid was removed by dialysis against excess of deionized water with frequent changes for 24 h at 277 K. A control experiment with the apo-forms of the LF fragments was also included to demonstrate that the differences in absorbance is an iron-specific effect and not caused by altering the pH.

### Antimicrobial assay of LF and its fragments

#### Antibacterial activity

The antibacterial assay was performed *in vitro* by the macro broth dilution method as described by Nonnecke and Smith et al. [40]. The efficacy of LF and its three fragments were expressed in terms of Lethal Concentration (LC_50_) which is described as the concentration of molecule (µg/ml) resulting in 50% reduction in the viability of the microorganisms as compared with that of the control. *E. coli* (EDL 933) and *S. pyogenes* M49 were cultured in 2% peptone water and Todd Hewitt broth respectively at 37°C and maintained with 1.7% w/v agar. *Y. enterocolitica* 8081 was grown in Luria Bertani (LB) broth and incubated overnight at 28°C, maintained with 1.7% w/v agar. *L. monocytogenes* (MTCC 839) was grown in Brain Heart Infusion broth at 37°C and maintained with Muller Hinton agar plates. Each strain was sub-cultured in fresh media and the absorbance at 600 nm was adjusted to 0.1. Antibacterial activities of the LF fragments were determined by microassay performed in sterile 96-well tissue culture plates (Nunc). Each well contained 100 µl of growth medium along with 10 µl of working inoculums and required volume of protein/protein fragment solution and incubated for 2 h. The final volume was made up to 200 µl using sterile water. Sterile medium was used as blank. Sterile water was added in place of the protein/protein fragment solution for negative control. Absorbance of all the samples at 600 nm was recorded for the entire concentration range (64 µg/ml–2048 µg/ml) increasing in a two-fold manner. All the experiments were performed three times in duplicates for all the individual fragments tested.

#### Disc diffusion assay

Disc diffusion assay was performed as described by Kirby-Bauer disk diffusion susceptibility test [41]. The bacterial strains were inoculated in respective broths and grown overnight at 37°C. Cells were then washed thrice with distilled water and approximately 10^5^ cells/ml were inoculated into molten agar-media at 42°C and poured into 100 mm diameter Petri dishes. After the bacterial colony had developed, sterile filter paper discs were placed on the plates distinctly. 50 µl of aliquot containing 20 mg/ml of protein/fragment solutions were added to the discs. Kanamycin (1 mg/ml) was also applied to the discs to serve as a positive control. The zones of inhibition (ZOIs) were observed after an incubation period of 48 h at 37°C.

#### Antifungal assay

Fungal strain of *C. albicans* (ATCC 90028) was used for evaluating antifungal activity of protein. The strains were grown in a medium containing (w/v) 1% yeast extract, 2% peptone and 2% dextrose (YPD medium), maintained on YPD plates with 2.5% w/v agar and the log phase mycelial suspensions incubated with LF and its fragments for 2 h. LC_90_ is described as the concentration of protein/protein fragment (µg/ml) resulting in 90% reduction in viability of the microorganisms determined *in vitro* by the macro broth dilution method.

#### Disc diffusion assay

Disc diffusion assay was performed as discussed by Ahmad et al. [42]. The fungal cells were inoculated in liquid YPD medium and grown overnight at 37°C. Cells were then washed thrice with distilled water and approximately 10^5^ cells/ml were inoculated into molten YPD agar at 42°C and poured into 100 mm diameter Petri dishes. After the mycelial colony had developed, sterile filter paper discs were placed distinctly on the plate. 50 µl aliquot of 20 mg/ml of protein solutions were added to the discs. Fluconazole (1 mg/ml) was also added to one of the discs to serve as a positive control. The ZOIs were observed after an incubation period of 48 h at 37°C. The size of halo formed indicates the fungicidal/static characteristic of the respective test compound [43].

## Results

### Preparation and purification of trypsin digested molecules of LF

The hydrolyzate of LF with trypsin showed five major bands, corresponding to 80 kDa, 45 kDa, 38 kDa, 21 kDa and below 14 kDa. The 80 kDa corresponded to the undigested LF while the <14 kDa band consisted of low molecular weight peptides which were generated on hydrolysis. The major fragments of LF which corresponded to bands at 45 kDa, 38 kDa and 21 kDa were designated as 45LF, 38LF and 21LF respectively.

**Figure 1:**
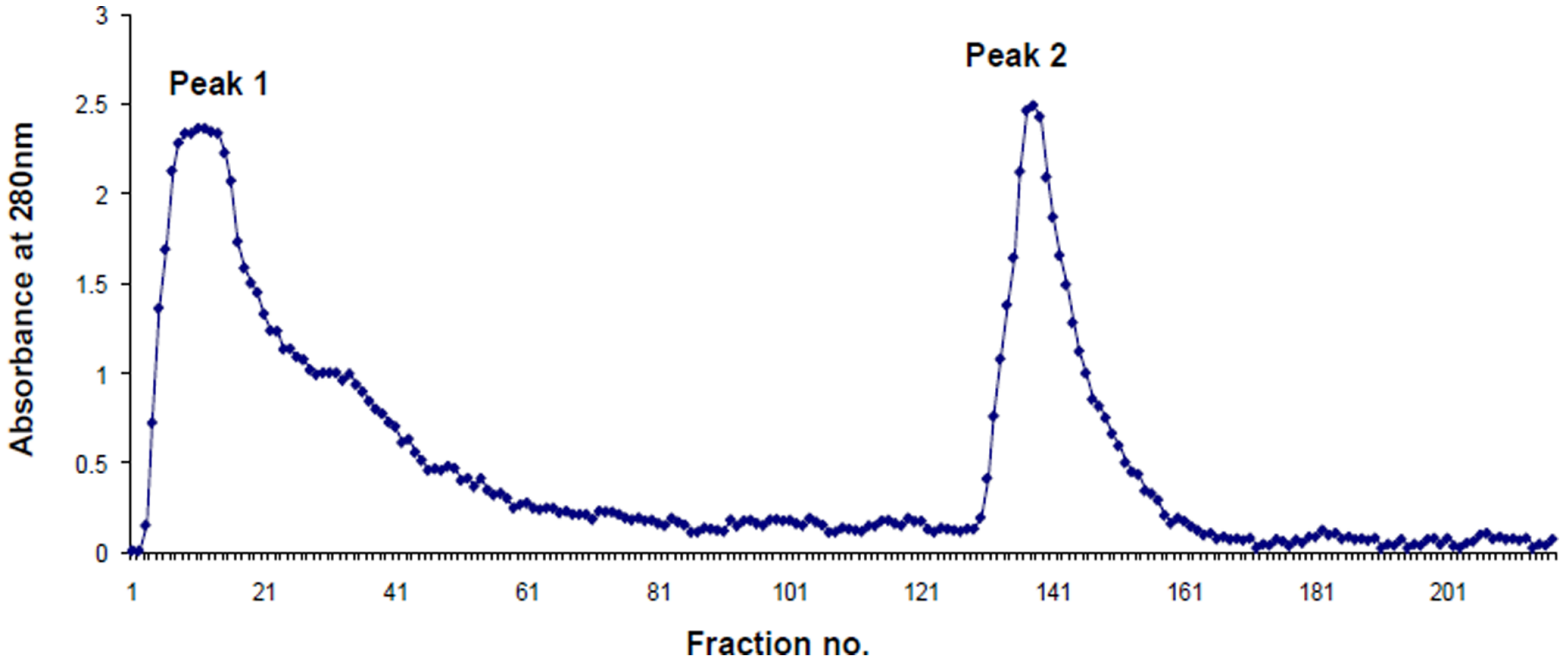
Elution profile during the purification of the hydrolyzate of bovine LF after trypsin digestion. Elution was performed using ion exchange chromatography by CM-Sephadex C-50 in 0.05 M Tris-HCl, pH 8.0. Peak 1 indicates the unbound fraction while Peak 2 is eluted in the bound fraction.

**Figure 2:**
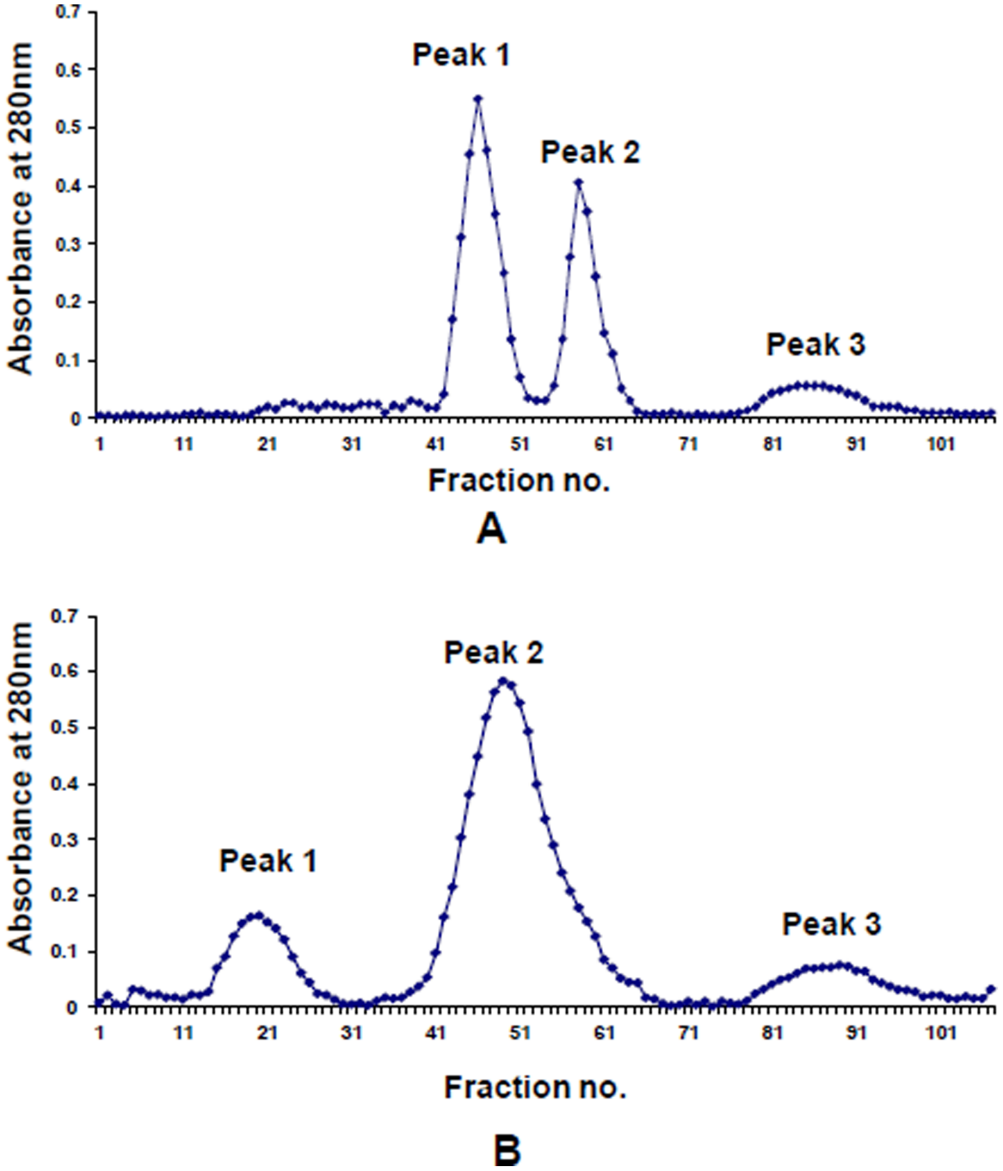
Elution profile of the unbound and bound fractions of tryptic digested bovine LF using gel filtration chromatography. (A) The unbound fraction yielded Peak 1 corresponding to purified 38LF fragment, Peak 2 corresponding to purified 21LF and Peak 3 corresponding to low molecular weight peptides. (B) The bound fraction yielded Peak 1 containing undigested LF, Peak 2 containing purified 45LF and Peak 3 corresponding to low molecular weight peptides.

The three functional fragments were purified using ion exchange and gel filtration chromatography. The LF hydrolyzate was applied on an ion exchange column of CM Sephadex C-50 using a salt gradient of NaCl (0.0–0.5 M) in 50 mM Tris-HCl (pH 8.0) (Fig. 1). Two fractions, unbound and bound were designated as Peak 1 and Peak 2 respectively. The unbound fraction contained the two fragments, 38LF and 21LF while the bound fraction contained the undigested LF and 45LF. The unbound fraction was further purified using gel filtration chromatography with Sephadex G-75, which yielded three peaks (Fig. 2A). While Peak 1 and Peak 2 corresponded to the purified fractions of 38LF and 21LF respectively, Peak 3 corresponded to low molecular weight peptides. Similarly, bound peak was fractionated using gel filtration chromatography with Sephadex G-75, which yielded three peaks (Fig. 2B). While Peak 1 was found to be undigested LF, Peak 2 and Peak 3 corresponded to 45LF and low molecular weight peptides respectively.

### N-terminal sequencing

**Figure 3:**
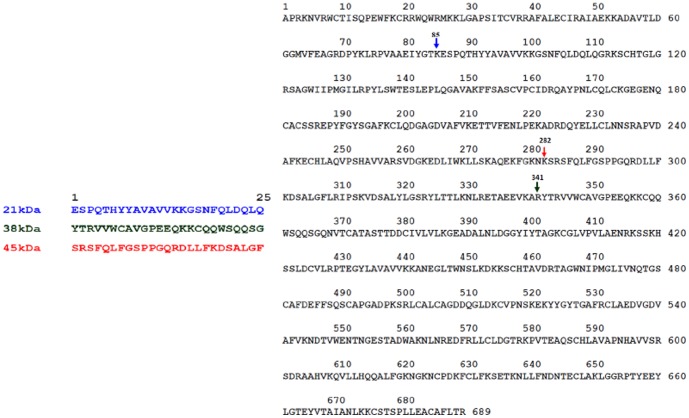
The entire sequence of bovine native LF. Arrows [blue (21LF), green (38LF), red (45LF)] indicate the origin sites of the three fragments respectively. The amino acid sequence of the first fifteen residues of 21LF, 38LF and 45LF is indicated against their names towards the left,

The N-terminal sequences of first 15 residues of the three LF fragments were obtained using PPSQ 21-A protein sequencer in order to identify them. The determined sequences of all the three molecules were identified in the native LF, the start point of which is indicated by arrows (Fig. 3).

### Sequence and structure analysis of proteolysis sites

**Figure 4:**
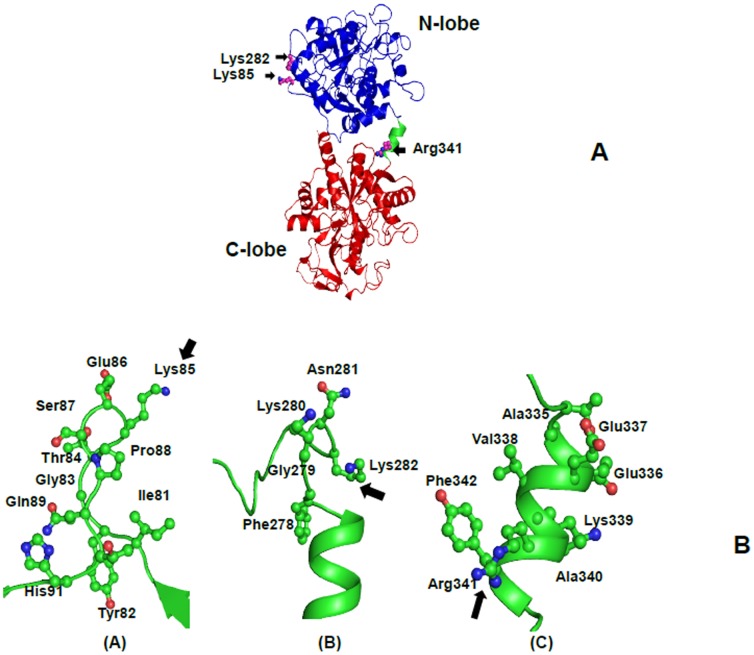
Schematic diagram of the bovine LF molecule [PDB code: 1BLF] and the three exposed loops of bovine LF. (A) The entire bovine LF molecule is shown. the N-lobe is colored in blue while the C-lobe is colored in red. The interconnecting helix connecting the two lobes is colored in green. The three sites at which trypsin cleave LF is indicated in black arrows against the residues which are targeted. (B) The three exposed loops of bovine LF which are targeted by trypsin to generate 21LF, 38LF and 45LF are shown and indicated with arrows against the residues which are cleaved (A) Lys85 (B) Lys282 and (C) Arg341.

**Figure 5:**
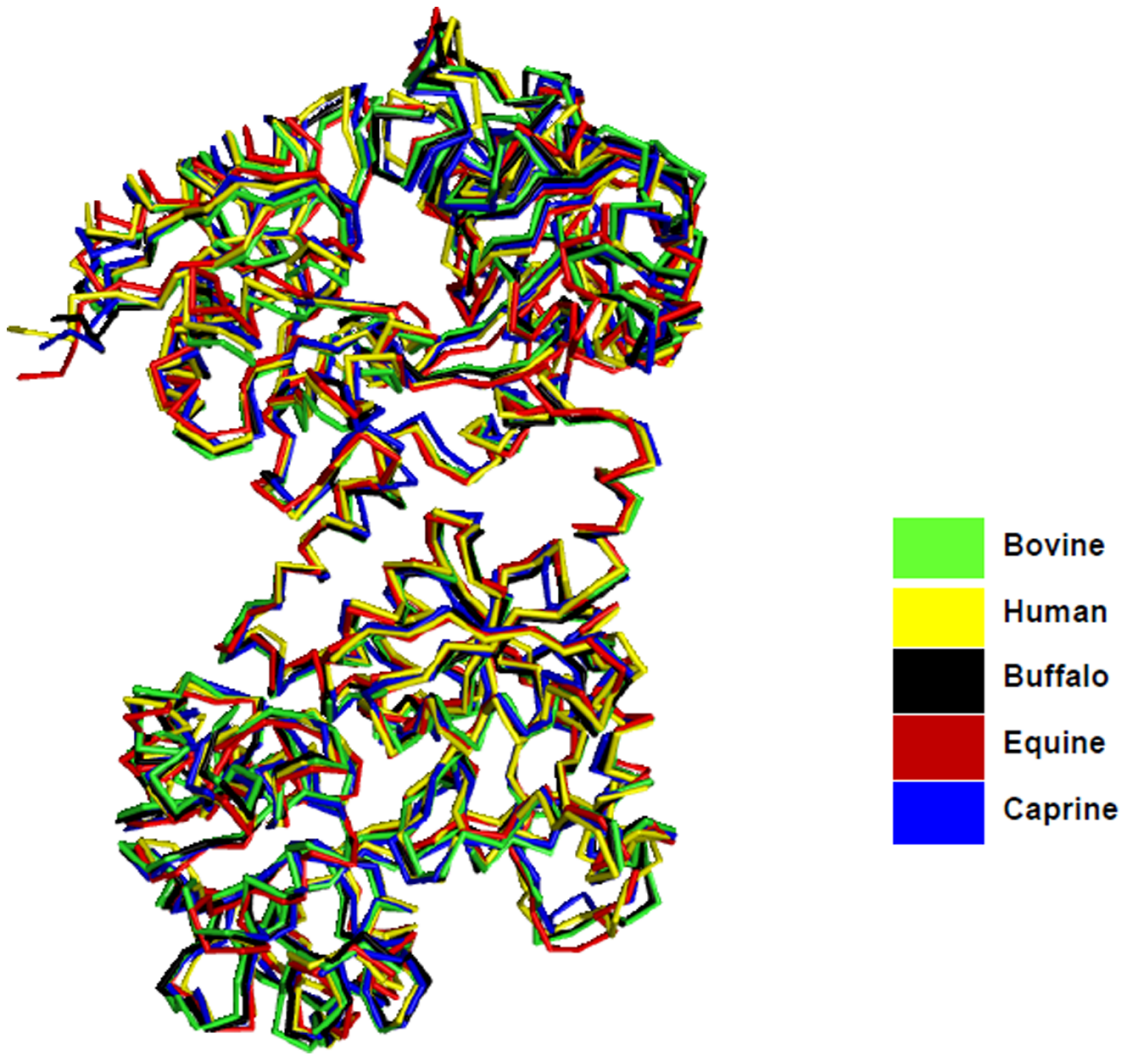
Superimposition of the backbone chains of iron-saturated LFs from various species. The figure shows that the overall chain folds in a similar fashion in all the species.

The N-terminal sequence determination of 21LF indicated that trypsin cleaves LF at Lys85, which is present in the loop Glu80-Tyr93. The 38LF is produced by hydrolysis at Lys281, which is present in the loop Phe278-Gly306. Similarly, 45LF is generated when trypsin cleaves LF at Arg341, which is situated on the helix which connects the N-lobe with C-lobe. It is significant to note that the three proteolysis sites are situated at exposed areas of LF (Fig. 4A and 4B). Also, in all the three sites, lysine and arginine are present which are the residues which are usually attacked by trypsin. While 21LF corresponded to the N2-domain and the 38LF corresponded to the C-lobe of the native LF, the 45LF corresponded to be a portion of the N1-domain attached to the C-lobe of native LF. Upon overlaying the backbone structures of iron-saturated LFs from different species, it is evident that they have a similar overall structure and folding (Fig. 5).

**Figure 6:**
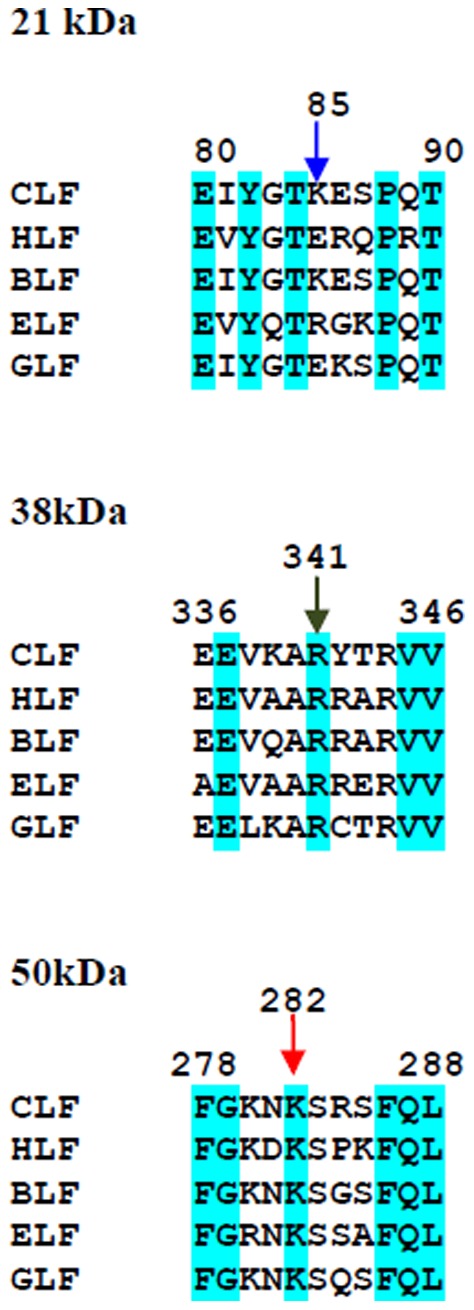
Sequence comparison of the cleavage sites of LF from various species. The different species are human (HLF), bovine (CLF), buffalo (BLF), equine (ELF) and caprine LF (GLF). The identity in the sequences is shown in blue. The places where trypsin cleaves the molecules are indicated by an arrow.

The first excision site is at Lys85, which is a residue that is conserved in buffalo LF (Fig. 6). The human lactoferrin (HLF) contains one extra amino acid at the N-terminus, and hence, the amino acid 85 becomes residue 86 in HLF which is arginine (Fig. 6). Similarly, the amino acid 85 in the case of equine Lf is arginine. Since arginine is attacked by trypsin as well, it seems that this site would be cleaved in all the above species. In case of caprine LF, though the 85^th^ residue is glutamine, the 86^th^ residue is lysine, which is exposed on the surface. Hence it may be possible that in the case of caprine LF, it may be the 86^th^ residue that serves as the excision site for trypsin. The second excision site is at Lys281, which is conserved in all the LFs. The third site of excision of LF by trypsin is at Arg341 which is present in the 3_10_-helix that connects the two lobes of LF. This arginine residue is also strictly conserved among LFs from all species. Incidentally, this is also the excision site for proteinase K [19], which obviously means that due to this site being the most exposed site in LF, this site would be the most labile site in all LFs and hence, would get cleaved by all proteases.

### pH induced release of iron from LF and its trypsin cleaved molecules

The native LF molecules contain two iron-binding sites, one in each lobe. However, it was expected that 38LF and 45LF would contain only one iron-binding site and 21LF would not have any iron-binding site, as the iron-binding cleft is situated in the interdomain cleft and 21LF is essentially a domain.

**Figure 7:**
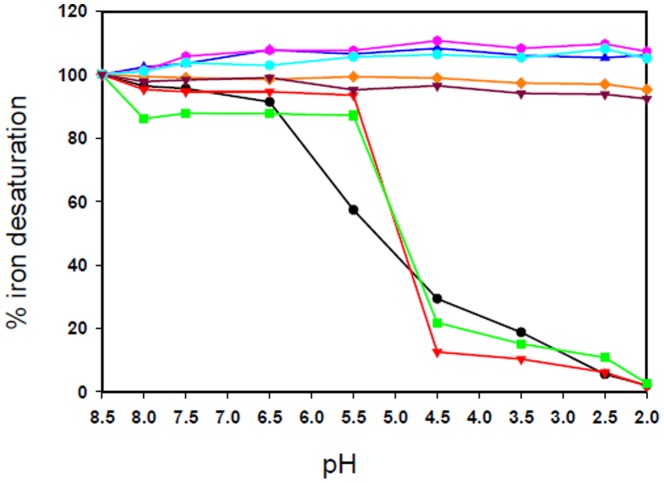
Comparative study of iron-desaturation of LF and its three tryptic fragments. The iron desaturation was plotted against decrease in pH for iron saturated bovine lactoferrin (black), 21LF (orange), 38LF (green) and 45LF (red) and their corresponding apo form of bovine lactoferrin (blue), 21LF (brown), 38LF (pink), 45LF (cyan) under identical conditions.

The results of comparative study of the desaturation of LF, 21LF, 38LF and 45LF are shown in Fig. 7. LF retained its iron till pH 6.5, below which it started losing iron steadily over a pH range of 6.5–2.0. The fragment 21LF did not show any change in iron saturation with change in pH, which indicated that this molecule had lost its iron binding site upon hydrolysis. In the cases of both 38LF and 45LF, the release of iron was almost identical, though it varied from that of the native LF. In 38LF and 45LF, the iron release started at pH 5.5, which was at a later point as compared to LF. These results were in accordance with the iron desaturation studies done on the proteinase K cleaved C-lobe of LF [19]. The two major reasons for the late iron-release in case of 38LF and 45LF may be due to the loss of inter-lobe interactions on hydrolysis as well as absence of the dilysine trigger made up of Lys210 and Lys301, which is present in case of N-lobe, but absent in C-lobe [44]. It is evident from these results that C-lobe needs the full N-lobe in order to release the iron at pH 6.5, as despite some inter-lobe interactions being present in 45LF, it still behaves like C-lobe in terms of iron release. No change was observed in pH induced iron release in any of the apo-forms of the molecules indicating that the differences in absorbance is an iron-specific effect and not caused by altering the pH.

### Antimicrobial activity of LF, 21LF, 38LF and 45LF

#### Antibacterial activity of LF, 21LF, 38LF and 45LF

**Figure 8:**
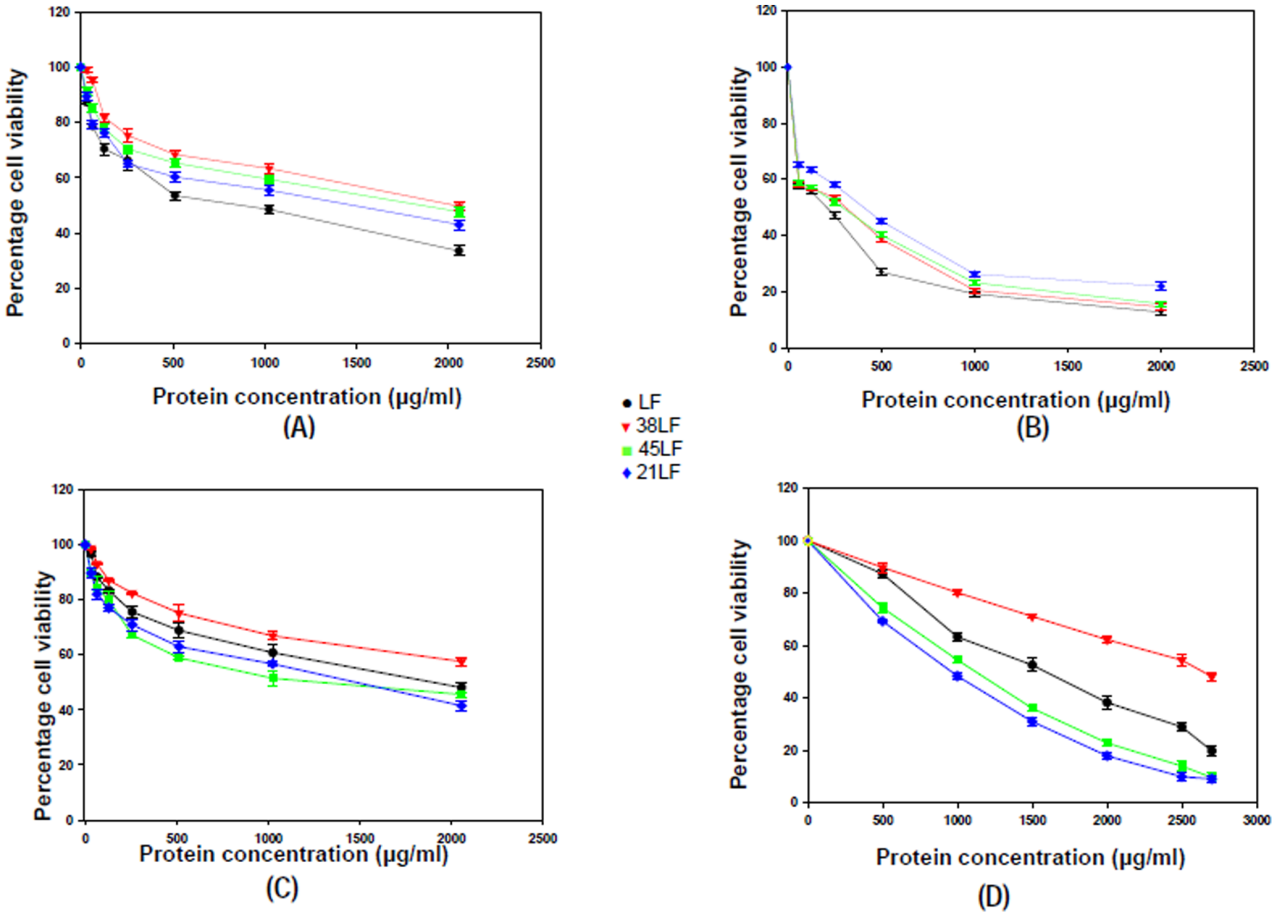
Antibacterial activity of LF and its three tryptic fragments. The antibacterial activity of LF (black), 21LF (blue), 38LF (red) and 45LF (green) has been plotted against (A) *S. pyogenes* M49 (B) *L. monocytogenes* MTCC 839 (C) *E. coli* EDL 933 and (D) *Y. entercolitica* 8081. All the data were expressed as mean values ± standard deviations; *P*<0.05, student's *t-* test.

The four molecules, LF, 21LF, 38LF and 45LF were tested on both gram positive bacterial strain (*S. pyogenes* M49 and *L. monocytogenes* MTCC839) and gram negative bacterial strain (*E. coli* EDL 933 and *Y. enterocolitica* 8081). The LC_50_ values of all the protein molecules were determined *in vitro* by the micro broth dilution method and given in Table 1. The data were expressed as mean values ± standard deviations. The statistical differences in the results were evaluated by student's t-test. The antibacterial activities of the LF molecules were further plotted in terms of percentage cell viability with increasing protein concentration (Fig. 8). In case of *S. pyogenes*, LF showed very high antibacterial activity with a LC_50_ (890.0±10.0) µg/ml as compared to 21LF, 38LF and 45LF with LC_50_ values of (1460.0±91.0) µg/ml, (2000.0±73.0) µg/ml, (1700.0±56.0) µg/ml respectively (Fig. 8, Table 1). In case of *L. monocytogenes* the LC_50_ values of LF, 21LF, 38LF and 45LF were found to be (200.0±2.6) µg/ml, (401.0±5.3) µg/ml, (290.0±1.8) µg/ml and (327.1±4.1) µg/ml respectively, showing that LF is the most effective against this bacteria.

**Table 1:** LC_50_ values of LF, 21LF, 38LF and 45LF against bacterial species.

	LF		21 kDa		38 kDa		45 kDa	
Bacteria	µg/ml	µM	µg/ml	µM	µg/ml	µM	µg/ml	µM
***S. pyogenes***	890.0±10.0	11.2±0.1	1460.0±91.0	69.5±0.4	2000.0±73.0.0	52.6±0.1	1700.0±56.0	37.7±0.1
***L. monocytogenes***	200.0±2.6	2.5±0.03	401.0±5.3	19.1±0.2	290.0±1.8	7.6±0.04	327.1±4.1	7.1±0.09
***E. coli***	1900.0±44.0	23.7±0.5	1460.0±31.0	69.5±1.4	>2000*±51.0	>52.6*±1.3	1400.0±43.0	31.1±0.9
***Y. enterolitica***	1500±81.0	18.75±1.01	900.0±27.0	42.8±1.2	2700.0±63.0	71.0±1.6	1100.0±78.0	22.0±1.5

All the data were expressed as mean values ± standard deviations; *P*<0.05, student's *t-* test.

*Highest concentration tested.

In case of gram negative bacteria, the LC_50_ of LF against *E. coli* was (1900.0±44.0) µg/ml. The same values for the other three molecules, 21LF, 38LF and 45LF, were found to be (1460.0±31.0) µg/ml, (>2000.0±51.0) µg/ml and (1400.0±43.0) µg/ml respectively against the bacterial strain. Similar results were obtained in case of *Y. enterocolitica* where 21LF and 45LF with LC_50_ values of (900.0±27.0) µg/ml and (1100.0±78.0) µg/ml respectively were found to be more effective in inhibition of bacterial growth than LF with LC_50_ of (1500.0±81.0) µg/ml. 38LF had minimal potency against *Y. enterocolitica* with LC_50_ of (2700.0±63.0) µg/ml.

**Figure 9:**
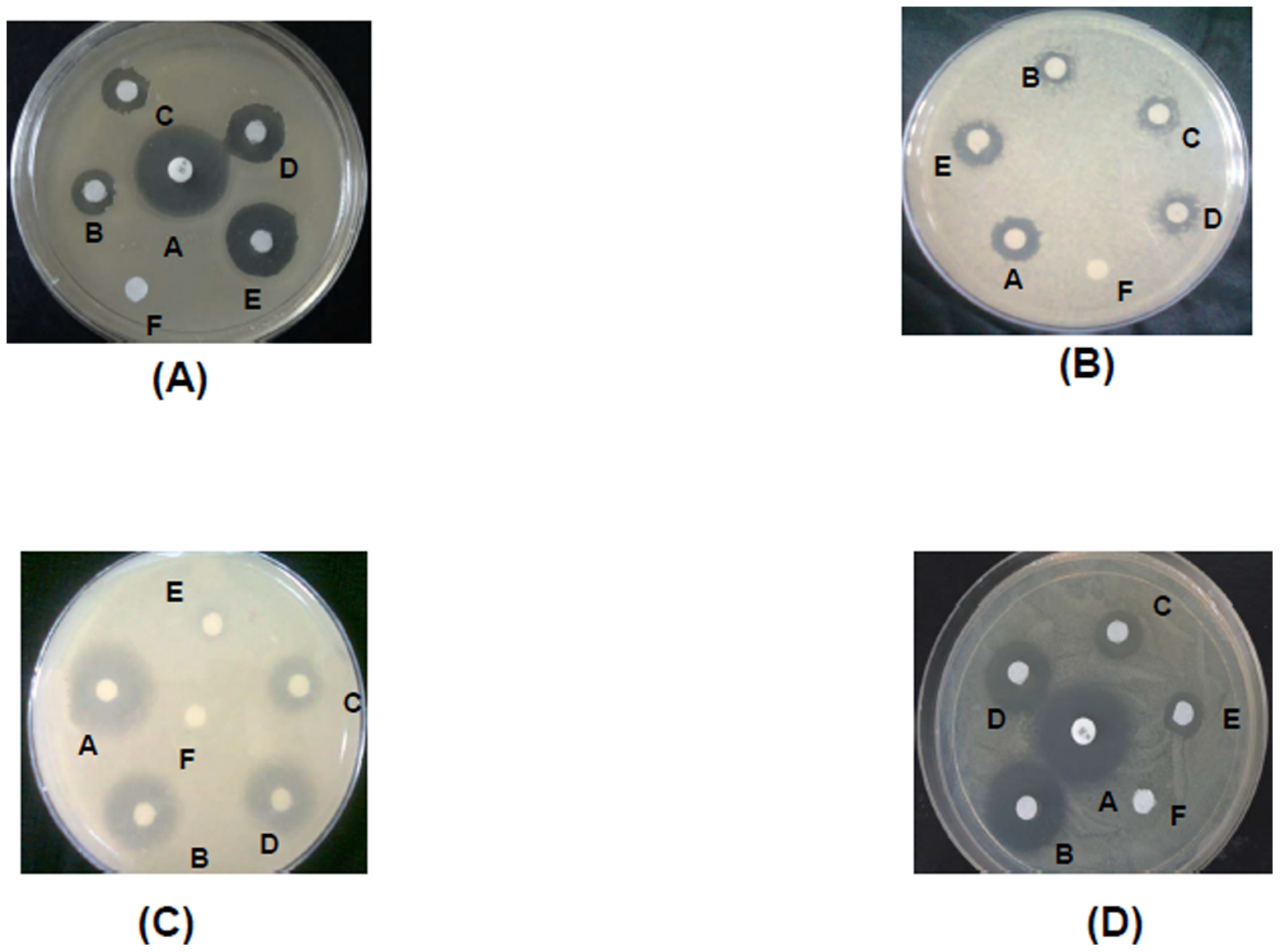
Zone of inhibitions against different bacterial species. The bacterial species used are (A) *S. pyogenes* M49 (B) *L. monocytogenes* MTCC 839 (C) *E. coli* (EDL 933) (D) *Y. enterocolitica* 8081. A corresponds to Kanamycin (positive control), B corresponds to 21LF, C corresponds to 38LF, D corresponds to 45LF, E corresponds to LF and F corresponds to blank.

The data were further confirmed by ZOIs observed in disc diffusion assay (Fig. 9). As shown in the figure, A, B, C, D and E are ZOIs by Kanamycin, 21LF, 38LF, 45LF and LF respectively. The diameters of zone of inhibition were listed in Table 2. Kanamycin is the positive control showing maximum inhibition in all three cases. In gram positive bacteria, ZOI of LF was more than the three fragments but in gram negative bacteria, 21LF had the largest diameter for ZOI showing its maximum potency against both the bacterial strains.

**Table 2:** Zone of diameters of LF, 21LF, 38LF and 45LF against bacterial species.

Proteins	*S. pyogenes* (mm)	*L. monocytogens* (mm)	*E. coli* (mm)	*Y. enterolitica* (mm)
**Kanamycin**	25	16	24	26
**LF**	19	14	6	9
**21LF**	15	9	21	20
**38LF**	13	10.5	12	13
**45LF**	11	11	16	17

Thus, *in vitro* studies show that the test compounds significantly inhibit growth of the bacteria, however, the potency of 21LF and 45LF were found to be higher than the whole protein. The potency of 38LF was found to be comparatively lower than all the other three molecules.

#### Antifungal activity of LF, 21LF, 38LF and 45LF

**Figure 10:**
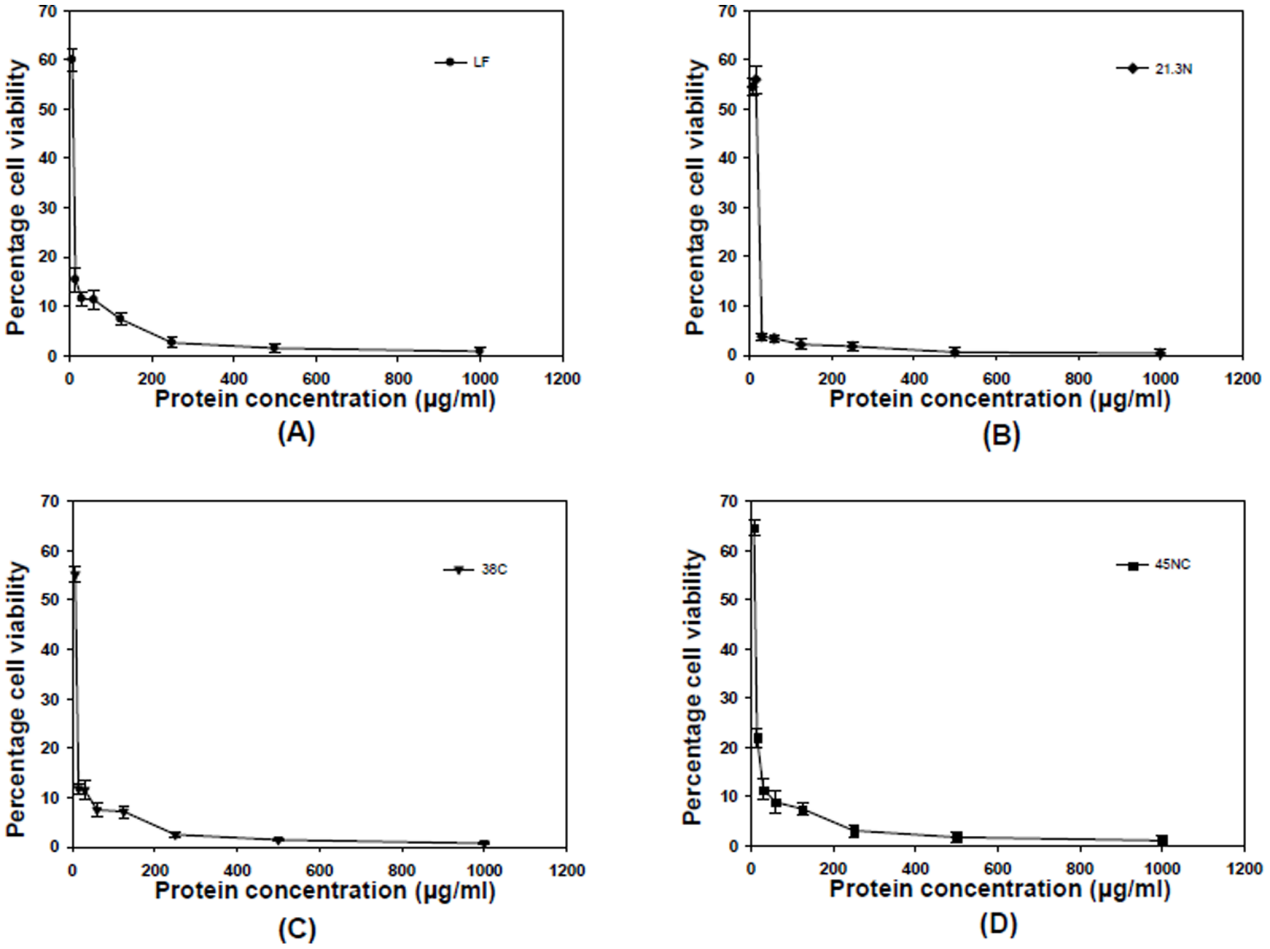
Antifungal activity of LF and its three tryptic fragments. The antifungal activity of (A) LF (•), (B) 21LF (♦), (C) 38LF (▾) and (D) 45LF (▪) is plotted against *C. albicans* ATCC 90028. All the data were expressed as mean values ± standard deviations; *P*<0.05, student's *t-* test

**Figure 11:**
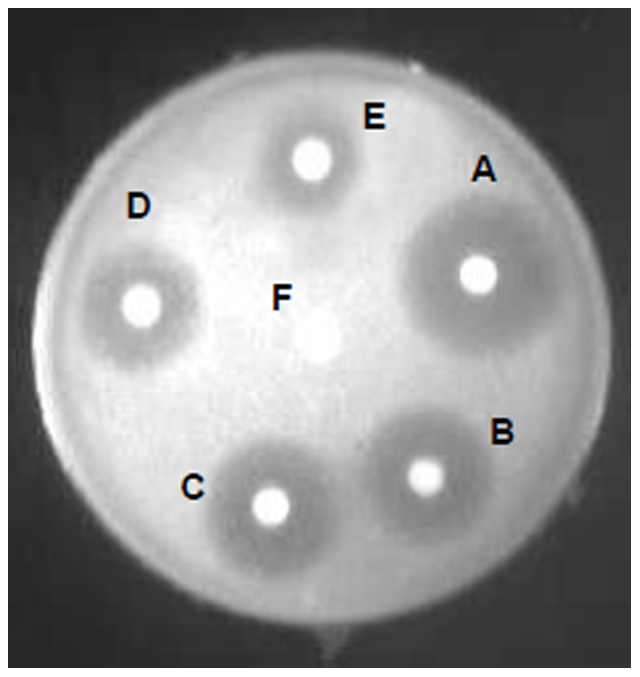
Zone of inhibitions against *C. albicans* ATCC 90028. A corresponds to Fluconazole (positive control), B corresponds to 21LF, C corresponds to 38LF, D corresponds to 45LF, E corresponds to LF and F corresponds to blank.

The antifungal activities of LF, 21LF, 38LF and 45LF are shown in Fig. 10. The LC_90_ values of the proteins obtained against *C. albicans* are listed in Table 3. The data were expressed as mean values ± standard deviations. The statistical differences in the results were evaluated by student's *t*-test. All the three fragments were found to exhibit more antifungal activities than the native molecule. 21LF with LC_90_ of (25.12±1.98) µg/ml had maximum activity against *C. albicans*, followed by 38LF with LC_90_ of (40.32±2.30) µg/ml and 45LF with LC_90_ of (47.52±2.51) µg/ml. The same value for the native protein was much higher with LC_90_ of (80.85±1.79) µg/ml. The data were further confirmed by ZOIs observed in disc diffusion assay (Fig. 11). As shown in the figure, A, B, C, D and E are ZOIs by Flucanozole, 21LF, 38LF, 45LF and LF respectively with corresponding diameters as 25 mm, 20 mm, 17 mm, 13 mm and 7 mm (Table 4). These data indicate that the antifungal activities of all the three fragments are substantially more than that of native LF. In this case, even 38LF showed a higher potency than the native molecule.

**Table 3:** LC_90_ values of LF, 21LF, 38LF and 45LF against *Candida albicans*.

	LC90	
Fragments	µg/ml	µM
LF	80.85±1.79	1.01±0.02
21 kDa	25.12±1.98	1.19±0.09
38 kDa	40.32±2.30	1.06±0.06
45 kDa	47.52±2.51	1.05±0.05

All the data were expressed as mean values ± standard deviations; *P*<0.05, student's *t-* test.

**Table 4:** Zone of diameters of LF, 21LF, 38LF and 45LF against *Candida albicans*.

Test Compound	Diameter of Zone of inhibition (mm)
**Flucanozole**	25
**LF**	7
**21LF**	20
**38LF**	17
**45LF**	13

## Discussion

LF is an antimicrobial protein which undergoes enzymatic digestion in the gut which results in the generation of its protein fragments and peptides. In order to maintain the antimicrobial functions of the protein, the hydrolyzed fragments should also display antimicrobial properties for the protection of neonates against the invading pathogens.

In this study, we have demonstrated the possible fate of bovine LF in the gut upon exposure to a major gut protease, trypsin. It is evident that bovine LF is hydrolyzed into three major functional fragments, 21LF corresponding to the N2 domain, 38LF corresponding to the C-lobe and 45LF, corresponding to the N1 domain attached to the C-lobe. Upon sequence analysis of the three proteolysis sites of LF, it is clear that these sites are conserved among all species. The iron desaturation study on these molecules shows that while 21LF binds no iron, the other two molecules, 38LF and 45LF show a similar iron-desaturation behavior, which is different from native LF. This indicates the entire N-lobe needs to be attached to C-lobe in order to release iron at pH 6.5, which is the norm in case of native LF.

The antimicrobial activities of bovine LF and its fragments 21LF, 38LF and 45LF against bacteria (*S. pyogenes, L. monocytogenes*, *E. coli, Y. enterocolitica*) and fungus (*C. albicans*) were found to variable. The antibacterial activity of LF against the gram positive bacteria, *S. pyogenes* and *L. monocytogenes* was higher than that of its fragments, 21LF, 38LF and 45LF. However, in case of gram negative bacteria (*E. coli* and *Y. enterocolitica*), the maximum antibacterial activity was observed in case of 21LF followed by 45LF. 38C was less potent as an antibacterial agent compared to the whole protein and the other fragments. On the contrary, antifungal activity of all the three fragments, including 38LF, was substantially higher than the native protein.

It may be noteworthy to observe that the initial portion of the N1-domain, which contains the membrane-penetrating, highly potent antibacterial peptides, has been excised away, most likely, into small peptides. Hence, this is the first study on the antimicrobial role of the other proteolyzed molecules, which contain the latter half of N1 domain, the entire N2 domain and C-lobe. Though 21LF does not have any iron binding capacity, it had the highest antibacterial activity compared to the other molecules. It has been established that segments of LF are strongly antibacterial using mechanisms of action which are distinct from iron-sequestration. These mechanisms could be binding and damaging the bacterial membrane [45]–[47] or synergistically interacting with components of the immune system in order to modulate and mount the immune response [48], [49]. Hence, it is assumed that 21LF would be exerting its antimicrobial activity using a mechanism of action other than iron-sequestration.

It had been noted in the past that hydrolysis of LF with trypsin did not have any significant effect on the antimicrobial activity or iron binding capacity of lactoferrin [23]. However, in that study, the entire hydrolyzate was used to estimate the antimicrobial activity. The fragments that were generated were not purified and their antimicrobial activity and iron-binding capacity were not estimated individually. In the present study, we have characterized the functional LF fragments which are generated due to hydrolysis of LF with trypsin. Since LF is mostly hydrolyzed into high molecular weight fragments, two of which retain the iron-binding capacity and all three retain the antimicrobial activity, it is now clear that LF as an antimicrobial defence molecule, has evolved to resist complete hydrolysis and breakdown of its activity.

Since this study as well as several related studies have concluded that LF does not lose its activity upon exposure to gut proteases, it would be interesting to speculate about the fate of LF upon exposure to microbial proteases of the microbiota. It has been reported in the past that *Vibrio cholerae* non-O1 protease cleaved LF into two fragments at Ser420, leading to the generation of two fragments corresponding to molecular weights of 50 kDa and 34 kDa [50]. Also, it was noted that the hydrolysis of LF with this proteases did not reduce the anti-bacterial activity of LF. In yet another related study, LF was upon incubation with growing *Streptococcus pneumonia*, got nicked at position Val78, leading to the generation of a LF fragment of molecular mass of about 8.6 kDa. This LF fragment corresponded to the highly antimicrobial N-terminal region of LF, and was responsible for killing the bacterial colonies [51]. Hence, it is obvious that LF as a potent antimicrobial agent has equipped itself to combat microbial as well as physiological gut proteases, so that upon exposure to all proteases, the cleavage products of LF are able to retain their antimicrobial activities.

Thus, these findings suggest that despite the hydrolysis of bovine LF by trypsin, LF continues to function in a similar and sometimes more potent manner, in form of its hydrolyzed functional fragments. Since LF, the main component of milk is ingested by neonates and most adults, studies on potent LF fragments may give direction for design of novel antimicrobial agents for the future.
